# The details and entropy demons in a transmembrane allosteric machine

**DOI:** 10.1371/journal.pbio.3003485

**Published:** 2025-11-26

**Authors:** Ben F. Luisi

**Affiliations:** Department of Biochemistry, University of Cambridge, Cambridge, United Kingdom

## Abstract

In E.coli, three of the four Type VII ABC transporter systems have been structurally characterized. This Primer explores a recent study in PLOS Biology that reports the cryo-EM structures of the fourth Type VII system, YbbAP-TesA, and suggests that YbbAP has a role in extracting hydrophobic compounds from the bacterial inner membrane.

## Teaser

In the model gram-negative bacterium *Escherichia coli*, three of the four Type VII ATP-binding cassette (ABC) transporter systems have been well characterized structurally and functionally. A new study reports the cryo-EM structures of the fourth Type VII ABC system, YbbAP-TesA, and provides evidence for its role in extracting hydrophobic compounds from the bacterial inner membrane and their subsequent hydrolytic transformation.

Allostery is a wonderfully powerful, yet deeply mysterious “Second Secret of Life.” It has continued to captivate the imagination of biochemists for decades and is conceptually straightforward: conformational changes induced by ligand binding at one site can be relayed throughout a molecule to impact changes at a distant site [1]. Yet how exactly this process occurs at the level of stereochemistry and eventually translates itself into complex changes in cellular behavior, remains incompletely understood. Hemoglobin remains one of the best-characterized examples of transport facilitated by allostery, linking molecular-level changes to organism-wide effects on oxygen delivery [2]. But what about ATP-activated membrane proteins? Could these less-well-understood transporters provide new insights into the mechanisms of allostery and potential cooperativity? In this issue, a study by McAndrew and colleagues suggests how a subclass of ATP-binding cassette (ABC) transporters can couple the energy of ATP binding and hydrolysis with the delivery of substrates to the active site of a distal enzyme that has putative lipid hydrolase activity [3]. The delivery is through long-range conformational changes across the lipid bilayer, constituting the allosteric aspect of the system. Although the system does not appear to enhance the catalytic rate constants for the associated hydrolase enzyme, it likely accelerates substrate capture and delivery and may represent a form of chelate cooperativity.

**Fig 1:**
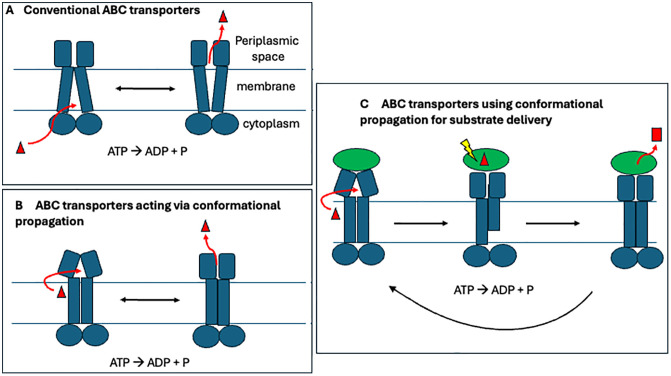
ABC transporters and the recruitment of a distal enzyme activity. The top panel left (A) is a simplified schematic of the conventional ABC transporters, posited to switch between inward- and outward-facing states. ATP binding and hydrolysis drives the conformational switch and resetting the system. The lower left panel (B) is a schematic for the group that communicate conformational changes across the membrane, but do not form channels to displace transport substrates. The right panel (C) depicts the action of the system studied by McAndrew and colleagues [3], where the conformational propagation helps to deliver substrates to a captive and orientated enzyme—in this case, a hydrolytic esterase.

The system examined by McAndrew and colleagues is the bacterial YbbAP complex, an ABC transporter belonging to a distinct subclass characterized by a signature organization of transmembrane helices. In conventional ABC transporters, the transmembrane helices accommodate and translocate ligands across the membrane through conformational changes that are coupled to, or in some cases, reset by, ATP binding and hydrolysis by the cytoplasmic ATPase domains [4,5]. The subset of this family to which the YbbAP complex belongs (the Type VII group) does not do the work of passing a molecule through the membrane. Instead, members of this class use ATP to drive numerous energetic processes on the extra-cytoplasmic side, including extraction of molecules from within the bacterial membrane or activation of periplasmic enzymes through conformational changes (Fig 1). Other Type VII transporters include MacB, which forms a tripartite assembly that spans the cell envelope and transports macrolides and small peptides, LolCDE, which uses ATP binding and hydrolysis to extract lipoproteins from the inner membrane and pass them to the periplasmic chaperone (LolA) for outer membrane delivery, and FtsEX-EnvC which allosterically regulates periplasmic amidases that cleave the peptidoglycan layer during cell division [6–8]. YbbAP-TesA differs from those other transporters in that it couples an ABC transporter with a lipid hydrolase (TesA) to extract and hydrolyze hydrophobic compounds from the membrane, establishing a new function for this transporter family.

The YbbAP transporter is a hetero-oligomeric assembly, comprising two cytoplasmic ABC domains (YbbA) and a transmembrane domain (YbbP) with an internal repeat, and the assembled machine bears pseudo 2-fold molecular symmetry. This complex stably binds to a single molecule of the hydrolase TesA, resulting in an overall asymmetric assembly that appears to extract hydrophobic molecules from the membrane for presentation to the hydrolase active site. As an annotated esterase, TesA has many potential substrates, but the physiological target is likely a lipid with one hydrocarbon chain (lysophospholipid). The reaction catalyzed by TesA might be a hydrolytic attack on a lysophospholipid to generate a fatty acid and glycerophospho-base.

What is the function of the YbbAP-TesA system, and why might it be allosteric? This question remains to be addressed, but there are some intriguing possibilities, such as the maintenance of membrane fluidity by hydrolysis of the lysophospholipids. Extracting hydrophobic compounds from the membrane may help in maintaining not only membrane integrity but also for detoxification or nutrient recycling. Intriguingly, there is no observable stimulation of TesA esterase activity in the YbbAP-TesA complex, nor any notable effect upon addition of ATP, suggesting the system does not allosterically activate the esterase. This is perhaps unsurprising, as esterase enzymes generally do not require an exogenous energy source like ATP to drive cleavage. Notably, the substrates tested in the study are soluble and readily accessible to TesA. However, in the context of the membrane, the extraction of substrates likely requires mechanical work, driven by conformational changes in the ABC component. There are parallels with the action of phospholipases, which extract substrates out of stable membranes [9], but those enzymes are generally ATP-independent and rely on surface activation upon enzyme binding to spontaneously drive substrate uptake into the active site pocket. While TesA may lack this capacity, its association with YbbAP makes up for this.

Reflecting on this system, questions naturally arise that may be relevant to all classes of ABC transporters. For instance, what prevents or modulates futile cycles of ATP hydrolysis? Could this regulation require some allosteric signaling, perhaps mediated by the TesA substrate in the case of YbbAP complex? Binding of the substrate or product may serve as a licensing step to the ATPase domains on the cytoplasmic side, which could be poised to fire and trigger changes that help to reset the system. It is also conceivable that the product of TesA action could influence local membrane fluidity, thereby modulating transporter conformation in a feedback loop. It is noteworthy that ester bond breaking is exothermic, and the esterase-catalyzed cleavage of a lysophospholipid could do work to drive conformational change [10]. It is also interesting to consider that this unique transporter-hydrolase architecture might be targeted for antibacterial drug development. Clearly, there are many avenues to be explored for this and related systems. Like Maxwell’s demon, which seemingly defies entropy by selectively arranging molecular states, the allosteric processes revealed in the study underpin events rich in meaningful biological information and showcase nature’s biochemical ingenuity.

## Abbreviation

ABC: ATP-binding cassette

## References

[1] Hunter CA, Anderson HL. What is cooperativity? Angew Chem Int Ed Engl. 2009;48(41):7488–99. doi: 10.1002/anie.200902490 19746372

[2] Perutz MF. Mechanisms of cooperativity and allosteric regulation in proteins. Q Rev Biophys. 1989;22(2):139–237. doi: 10.1017/s0033583500003826 2675171

[3] McAndrew M, Cook J, Gill A, Sahoo K, Thomas C, Stansfeld P. Structural characterization of the YbbAP-TesA ABC transporter identifies it as a lipid hydrolase complex that extracts hydrophobic compounds from the bacterial inner membrane. PLoS Biol. 2025;23(11):e3003427. doi: 10.1371/journal.pbio.3003427 PMC12646458 41289308

[4] Hofmann P, et al. Conformation space of a heterodimeric ABC exporter under turnover conditions. Nature. 2020;571:580–3. https://www.nature.com/articles/s41586-019-1391-0 10.1038/s41586-019-1391-0 PMC7612745 31316210

[5] Alam A, Locher KP. Structure and mechanism of human ABC transporters. Annu Rev Biophys. 2023;52:275–300. doi: 10.1146/annurev-biophys-111622-091232 36737602

[6] Bei W, Luo Q, Shi H, Zhou H, Zhou M, Zhang X, et al. Cryo-EM structures of LolCDE reveal the molecular mechanism of bacterial lipoprotein sorting in *Escherichia coli*. PLoS Biol. 2022;20(10):e3001823. doi: 10.1371/journal.pbio.3001823 36228045 PMC9595528

[7] Fitzpatrick AWP, Llabrés S, Neuberger A, Blaza JN, Bai X-C, Okada U, et al. Structure of the MacAB-TolC ABC-type tripartite multidrug efflux pump. Nat Microbiol. 2017;2:17070. doi: 10.1038/nmicrobiol.2017.70 28504659 PMC5447821

[8] Cook J, Baverstock TC, McAndrew MBL, Roper DI, Stansfeld PJ, Crow A. Activator-induced conformational changes regulate division-associated peptidoglycan amidases. Proc Natl Acad Sci U S A. 2023;120(24):e2302580120. doi: 10.1073/pnas.2302580120 37276423 PMC10268282

[9] Dennis EA. Allosteric regulation by membranes and hydrophobic subsites in phospholipase A2 enzymes determine their substrate specificity. J Biol Chem. 2022;298(5):101873. doi: 10.1016/j.jbc.2022.101873 35358512 PMC9079178

[10] Szöke A, Scott WG, Hajdu J. Catalysis, evolution and life. FEBS Lett. 2003;553(1–2):18–20. doi: 10.1016/s0014-5793(03)01008-1 14550539

